# Start Strong, Finish Strong: A Review of Prehabilitation in Cardiac Surgery

**DOI:** 10.3390/life14070832

**Published:** 2024-06-29

**Authors:** Vincent Bargnes, Steven Davidson, Lillian Talbot, Zhaosheng Jin, Jeremy Poppers, Sergio D. Bergese

**Affiliations:** 1 Department of Anesthesiology, Stony Brook University Hospital, Stony Brook, NY 11794, USA; 2 Renaissance School of Medicine, Stony Brook University, Stony Brook, NY 11794, USA

**Keywords:** cardiac prehabilitation, exercise, nutrition, psychobehavioral

## Abstract

Cardiac surgery constitutes a significant surgical insult in a patient population that is often marred by significant comorbidities, including frailty and reduced physiological reserve. Prehabilitation programs seek to improve patient outcomes and recovery from surgery by implementing a number of preoperative optimization initiatives. Since the initial trial of cardiac prehabilitation twenty-four years ago, new data have emerged on how to best utilize this tool for the perioperative care of patients undergoing cardiac surgery. This review will explore recent cardiac prehabilitation investigations, provide clinical considerations for an effective cardiac prehabilitation program, and create a framework for future research studies.

## 1. Introduction

Cardiac surgery is an ever-evolving field, with recent advancements including the use of endovascular interventions and intraoperative transesophageal echocardiography. We will focus our review on the role of preoperative rehabilitation (prehabilitation) in cardiac surgery. 

The premise of prehabilitation is to increase a patient’s functionality before undergoing surgery in order to mitigate the decline in the patient’s postoperative function. Further, the lessons patients learn from prehabilitation may help hasten their recovery and improve postoperative health. Historically, prehabilitation was not thought of as rehabilitation before surgery. In the 1940s, the British Army was one of the first organizations to use the term prehabilitation [1]. They discovered that additional support in the form of exercise, education, nutrition, and socialization before deployment improved the potential of recruits who were at risk of not performing to the Army’s standards. This concept of prehabilitation has since been applied to many surgical subspecialties, including colorectal, orthopedic, surgical oncologic, and cardiothoracic [2,3,4,5], as a way of preparing patients for surgery and subsequent recovery, just as prehabilitation prepared British troops for battle.

Enrollment in a prehabilitation program requires substantial interdisciplinary planning that may not be practical for all patients, especially those who require emergent surgical intervention. Patients with acute or decompensated cardiac, vascular, and pulmonary diseases also may not be eligible for prehabilitation due to the elevated cardiovascular risks associated with physically taxing programs. Additionally, patients with established cancer diagnoses require special consideration for prehabilitation, as enrollment may delay oncologic care and expose patients to elevated risks of metastasis. This review will explore the available literature on prehabilitation in cardiac surgery and provide guiding points for clinicians and researchers alike as we move forward to improve patient care and recovery following cardiac surgery. 

## 2. Frailty

Frailty is a clinical syndrome, marked by reduced strength, endurance, and physiological function, that significantly affects the recovery trajectory of a patient following cardiac surgery [6,7,8]. Patients who are frail experience a loss of physiological reserve, diminishing their ability to respond to and cope with physical and emotional stress. The pathophysiology of frailty involves functional decline across multiple body systems and presents as disruptions in homeostatic responses [9,10]. Frailty is exacerbated by advanced age, medical comorbidities, low physical activity, and poor nutrition. Although it is independent from the normal aging process, older individuals are highly susceptible to frailty. In patients undergoing surgery, frailty is correlated with a variety of poor postoperative outcomes [8,11,12,13,14]. Given that frailty has been hypothesized to be a result of a pro-inflammatory aging state, the pro-inflammatory state associated with cardiovascular disease (CVD) may help to explain why patients who require cardiac surgery would be at a high risk of frailty [15].

In the general population, 15% of adults 65 years or older meet the criteria for frailty, taking into account factors such as slowed walking time and poor grip strength, while up to 50% are considered pre-frail [6,10,16]. Among cardiac surgery patients, the overall prevalence of frailty is estimated to be between 4 and 10% and is higher in older patients, female patients, and those with comorbidities [7,12,17]. Poor post-surgical outcomes that are statistically correlated with frailty in cardiac surgery patients include increased mortality rates, increased hospital length of stay (LOS), increased intensive care unit (ICU) LOS, decreased postoperative functional status, higher rates of hospital readmission, and higher rates of major surgical complications, including sternal wound infection and stroke [7,8,12,17]. Similarly, in the general surgery population, frailty correlates with poor surgical outcomes [13,14]. In a large cohort of patients undergoing thoracic surgery, frailty was statistically correlated with pneumonia, prolonged intubation, and mortality [14]. The prevalence of frailty among cardiac patients and the role frailty plays in complicating recovery underscore the need for the integration of frailty assessments into preoperative evaluation to guide clinical decision-making [18]. Determining the extent of frailty present in patients preparing for cardiac surgery and exploring the potential for frailty reversal is one of the main goals of cardiac prehabilitation [19]. 

## 3. Components of Prehabilitation Programs

Prehabilitation may be especially important as people are living longer and presenting for cardiac surgical procedures with significant frailty and more extensive comorbid conditions. Physiological reserve is an important prognostic indicator commonly used to describe how a patient outcome, either positive or negative, is viewed in relation to a stressor, such as cardiac surgery [20]. Due to its complexity, there is no single diagnostic test to completely evaluate an individual patient’s reserve. 

Within the setting of total knee arthroplasty (TKA) and total hip arthroplasty (THA) for osteoarthritis, prehabilitation has been shown to significantly reduce the need for postoperative rehabilitation; however, there is limited research on the use of prehabilitation within the cardiac population [21]. Prehabilitation has often been compared to training for a marathon, grounded on evidence that structured and sustained lifestyle changes over time lead to cardiovascular, respiratory, and muscular conditioning [22]. The benefits of exercise, nutrition, and psychobehavioral interventions before surgery are well-established. 

### 3.1. Exercise Component

In frail patients undergoing cardiac surgery, reduced physical fitness and physical deconditioning before, during, and after surgery have large negative effects, thus increasing morbidity, mortality, and hospital LOS. If patients are robust enough to tolerate high levels of physiological stress before surgery, the patients are more likely to be able to tolerate the stress of surgery and recover to at least their preoperative functional capacity. Prehabilitation exercises, including aerobics, strength training, and inspiratory muscle training, strive to increase a patient’s physical fitness and functional capacity prior to heart surgery and can be assessed through proxy measurements such as the 6-min walk test, the Timed-Up-and-Go Test, and the MacNew quality-of-life (QoL) questionnaire [2]. Despite data showing that increased fitness is associated with decreased mortality, there remains a fear of physical activity among many patients who are apprehensive about beginning an exercise program prior to cardiac surgery [23,24]. In patients awaiting elective cardiac surgery, structured exercise programs are safe and may decrease postoperative complications and hospital LOS [2,25].

#### 3.1.1. Aerobic Exercise 

An array of aerobic physical exercise prehabilitation interventions have been assessed, including cycle ergometers and treadmills. Typically, these exercise interventions are prescribed over a two- to eight-week timeframe, with the suggestion that longer programs result in greater benefits [25]. The first randomized controlled trial (RCT) of preoperative aerobic exercise in patients undergoing elective coronary artery bypass graft (CABG) surgery was carried out almost 25 years ago. Patients deemed to be low-risk, as defined as nonvalvular cardiac surgery with a preoperative left ventricular ejection fraction of 40% or greater, were signed up for a twice-weekly supervised exercise program at an intensity of up to 70% of functional capacity for 90 min per session. In conjunction, they also received two supportive educational sessions during the same period, as well as a monthly call with the nurse clinician. This study found that patients in the intervention group had significantly shorter lengths of stay in the ICU and in the hospital and better physical functioning, as well as improved QoL both before and after surgery [26]. 

In a more recent RCT, participants awaiting elective CABGs and randomized in the intervention group were enrolled in a two-week supervised exercise program three times per week at an intensity of up to 70% peak oxygen uptake. Each session included two 10-min cycling workouts with 15 min of light gymnastics in between. The cycling workouts gradually increased to 25 min by the end of the trial. The changes in 6-min walk distance and Timed-Up-and-Go time were significantly greater in the intervention group compared with the control group in the preoperative period. These two assessments suggested that this prehabilitation improved physical fitness and functional capacity before incision. This group of patients with higher levels of fitness and functionality went on to experience greater postoperative improvements in physical fitness and functional capacity when compared with the control group [2]. Steinmetz et al. also demonstrated significant improvements in all domains of QoL after two-week prehabilitation; however, this did not meet the minimal clinically important difference. In a 2023 systematic review and meta-analysis of patients awaiting cardiac surgery, exercise-based prehab had a significant impact on functional capacity, as demonstrated by improved 6-min walk distances, significantly shorter hospital stays, and a reduced risk of postoperative atrial fibrillation in patients under 65 years of age [27].

#### 3.1.2. Strength Training

Strength training has an important role in prehabilitation programs, given that the surgical recovery period is often contradictory to maintaining, let alone improving, strength. Postoperative surgical states often limit mobility due to various postoperative surgical requirements, such as intubation, chest tubes, surgical drains, and vascular access. A patient with minimal strength before surgery may become deconditioned because of this immobility during recovery to a level that would predispose them to falls and subsequent complications that would prolong their recovery. 

Waite et al. investigated home-based strength and balance exercise training in their 22-patient, single-center observational study. The exercises included wall push-ups, heel raises, bicep curls, sit-to-stand, knee bends, backward walking, and tandem walking. Each exercise featured four levels of difficulty based on resistance band strength and repetitions. Before the home-based program began, each exercise was demonstrated for the participants, and the participants were observed performing the exercises. The exercises were individually tailored to each patient by a physiotherapist, and the progressive levels were developed from the Otago Exercise Program, which has been shown to reduce falls in patients who are older adults and frail. Waite et al. measured postoperative LOS and functional capacity with the Duke Activity Status Index, the Short Physical Performance Battery Protocol, and the 6-min walk test. Frailty status was evaluated using the clinical frailty scale measuring from ‘very fit’ to ‘terminally ill’. Similar to the prehabilitation aerobic exercise trials, their results demonstrated reduction in frailty, improved 6-min walk distance, and improved functional capacity [28].

#### 3.1.3. Inspiratory Muscle Training

A wide range of pulmonary complications following cardiac surgery occurs in a substantial proportion of patients, including atelectasis, pleural effusion, phrenic nerve injury, pneumonia, and acute respiratory distress syndrome (ARDS), resulting in variable levels of subsequent morbidity, mortality, and hospital LOS [29,30,31]. Especially in patients with chronic pulmonary conditions such as chronic obstructive pulmonary disease (COPD), improving respiratory muscle strength preoperatively has been a focused area for improving outcomes. Combined with incentive spirometry, deep-breathing and forced-expiration exercises have been shown to improve respiratory muscle strength and enhance sputum clearance. They are also simple and low-risk and can be performed at home. 

The first RCT of respiratory prehabilitation was performed in 2006, in which patients at high risk of postoperative pulmonary complications were enrolled in an 8-week program consisting of 20 min of incentive spirometry, forced-expiration, and deep-breathing exercises daily. This was carried out at home and supervised by a physiotherapist once a week. Compared with the control group, the intervention group had decreased incidences of pneumonia, pleural effusions, and need for re-intubation; however, there was no significant reduction in the rate of respiratory failure requiring prolonged ventilation [32]. Balandiuk et al.’s RCT compared 48 h of preoperative incentive spirometry to usual care and described lower rates of hypoxemia perioperatively in the intervention group as well as decreased mechanical ventilation time [33]. Two subsequent trials also found that respiratory prehabilitation may improve respiratory strength and reduce postoperative hospital LOS [34,35].

Aerobic exercise, strength training, and inspiratory muscle training are three interventions that belong to the exercise component of a prehabilitation program. These interventions have resulted in various clinical effects, which are summarized in Table 1 [36,37,38]. Despite differing degrees of medical supervision during exercise among the trials, all have been conducted safely in patients pending cardiac surgery. 

### 3.2. Nutrition Component 

Diet and nutritional status are established and modifiable risk factors for the development and progression of CVD and frailty [39]. Excessive calorie consumption contributes to chronic low-level inflammation, obesity, and hyperglycemia, which together drive the progression of CVD [40]. Conversely, malnutrition and sarcopenia correlate with increased mortality in CVD [41,42,43]. Patients undergoing cardiac surgery with low body mass indexes (BMIs) are more likely to experience postoperative complications and have higher rates of postoperative mortality than their normal-BMI counterparts [44,45,46,47]. Having a BMI between 30 kg/m^2^ and less than 40 is not associated with higher rates of mortality following cardiac surgery, and numerous studies have shown that there may be a protective effect of increased body mass in the perioperative period [48,49,50,51,52,53,54]. A classification of severe obesity or class III obesity, defined as a BMI of 40 or greater, is correlated with post-surgical complications including increased risk of sternal wound infection, renal failure, and prolonged intubation post-cardiac surgery [55,56]. This suggests that identifying and nutritionally fortifying underweight or malnourished patients before cardiac surgery may improve their postoperative trajectory. Validated screening tools to identify malnourished patients beyond the BMI include the Subjective Global Assessment (SGA), the Malnutrition Universal Screening Tool (MUST), and the Short Nutrition Assessment Questionnaire (SNAQ©) [57].

Guidelines specific to cardiac surgery from the Enhanced Recovery After Surgery Society recommend the correction of nutritional deficiencies and improvement of glycemic control before surgery in addition to preoperative exercise; however, they do not recommend a specific preoptimization regime, citing a lack of supportive evidence [58]. Multidisciplinary cardiac prehabilitation programs provide education on lifestyle modifications beyond physical conditioning. To our knowledge, only two published cardiac prehabilitation studies have included nutrition interventions. According to Hartog et al., patients randomized to the Heart-ROCQ program were assessed by a dietitian and received group education on healthy lifestyle modifications in addition to exercise training. Compared to the controls, the participation in Heart-ROCQ prehabilitation was correlated with lower rates of postoperative atrial fibrillation [59,60]. According to Sawatzky et al., the prehabilitation intervention group received 12 class-based education sessions, which included topics on diet. The prehabilitation group walked further distances immediately following surgery and three months after surgery when compared with the controls [38]. Isolating the effects of dietary counseling from the multidisciplinary approach on postoperative outcomes was challenging. Cheung et al. are currently enrolling patients in a cardiac surgery prehabilitation program where patients receive whey protein supplementation and weekly dietary counseling sessions prior to surgery [61].

Nutritional prehabilitation is well-studied in the context of gastrointestinal and oncological surgery due to the high prevalence of cachexia among these patients and is associated with reduced postoperative morbidity [62,63,64]. Targeting specific macronutrient goals, especially for protein, and providing protein supplementation in conjunction with exercise regimes may further improve postoperative outcomes. In studies of colorectal and esophageal cancer patients, those randomized to receive both individualized nutritional counseling with protein goals of 1.5 g/kg and oral protein supplementation as part of their multidisciplinary prehabilitation interventions had superior postoperative outcomes compared with the controls [3,5]. According to Carli et al., frail patients awaiting colorectal cancer resection received a similar macronutrient-targeted nutritional regime as part of a multidisciplinary prehabilitation intervention; however, no significant differences in postoperative complications or outcomes were observed [65]. Frail patients may require higher concentrations of macromolecules to reverse long-standing malnutrition and improve surgical outcomes. 

Pre-surgical micronutrient supplementation is a controversial topic that requires further investigation. According to Leong et al., cardiac surgery patients treated with coenzyme Q10, magnesium, lipoic acid, omega-3 fatty acids, and selenium had shorter hospital LOSs than placebo controls [66]. Meta-analysis of vitamin C supplementation in the cardiac surgery perioperative period was associated with a decreased incidence of atrial fibrillation, reduced ventilation time, and reduced LOS—both hospital and ICU stays [67]. In a trial of 78 patients, those treated 1 day preoperatively and 3 weeks postoperatively with vitamin E and zinc showed reduced hospital LOSs compared with the placebo patients. In a meta-analysis evaluating the efficacy of intravenous iron therapy in anemic patients, with both iron-deficiency anemia and other causes of anemia, awaiting cardiac surgery failed to show differences in postoperative outcomes or mortality between the treated and control groups [68].

Nutritional support is a complex component in prehabilitation that strays from the realm of pharmaceutical intervention modern American physicians are comfortable with, requiring physician collaboration with dietitians to educate patients on the importance of a balanced diet. Prehabilitation programs should equip patients with mechanisms to carry out nutritional prehabilitation, including employing the infrastructure of the increasingly popular meal preparation industry. 

### 3.3. Psychobehavioral Component 

The third component of prehabilitation relates to the broad array of psychological and behavioral interventions provided before major cardiac surgery, including addiction treatment and mental health optimization. One of the most heavily researched interventions within this component is smoking cessation. Smoking cigarettes couples the already deleterious effects of inhaling combusted material with exposure to addictive nicotine, resulting in potent habit formation and an important target for harm reduction. Smoking cessation requires a broad treatment perspective complete with an addiction medicine lens for a successful cessation campaign. While there is clear evidence to support that preoperative smoking cessation improves postoperative recovery, including respiratory sequelae and wound healing, there is also clear evidence that successful smoking cessation is multidimensional and deserves a robust care plan involving a combination of individual counseling, pharmacological assistance, and large-scale interventions that reduce systematic barriers to care for and shape societal factors against smoking [69,70,71]. While the optimal smoking cessation timeline has not been established and has been better covered by McCann et al., prehabilitation should provide early and physician-guided access to a multifaceted smoking cessation program, which may include psychosocial therapy, prescription cessation aids, and active participation in population-level efforts at curbing the impact of tobacco on communities [25]. 

The psychobehavioral component of prehabilitation focuses on mentally preparing a patient for a major surgery. Preoperative anxiety has been associated with higher mortality following cardiac surgery, encouraging perioperative teams to reduce anxiety well before the morning of a surgery [72]. The literature on this component exhibited remarkable variation between prehabilitation studies. Among those that included psychobehavioral interventions, each approached this component differently, from monthly nurse-initiated phone calls providing reassurance to a dozen classes on broad topics that included stress management to twice-weekly multidisciplinary sessions that included an emphasis on behavioral change to individualized hour-long sessions with family member involvement facilitated by an experienced occupational therapist specializing in stress reduction [26,36,38,73]. The studies on preoperative anxiety reduction exhibited different levels of benefit for patients and took place in various settings, including patients admitted to the hospital pending cardiac surgery and those who had several weeks at home before their cardiac surgery, creating difficulty comparing the effects of the different psychobehavioral interventions. 

Most published prehabilitation trials have focused on the effects of physical prehabilitation and lacked psychobehavioral components, and some trials have consisted of both, yet a limited third group has focused solely on the effects of psychobehavioral components. Dao et al.’s 2011 RCT belongs to this third group. They found that four 60-min sessions of cognitive behavioral therapy (CBT), including muscle relaxation and cognitive-restructuring techniques, for the treatment of depression and anxiety symptoms with a clinical psychologist improved depressive symptoms, anxiety symptoms, QoL, and in-hospital LOS [74]. This type of psychobehavioral intervention was novel in prehabilitation trials and remains so as of this writing. 

Selected postoperative behaviors, especially postoperative cardiac rehabilitation participation, are clinically important and can reduce major adverse postoperative cardiac and cerebrovascular events [75]. While the current data are not able to attribute cardiac rehabilitation participation to a specific prehabilitation component, two studies have detected a strong signal that patients undergoing prehabilitation programs consisting of nutrition, exercise, and psychobehavioral components have a higher rate of postoperative cardiac rehabilitation enrollment compared with patients receiving the current standard of care [38,73]. Given the importance of cardiac rehabilitation, prehabilitation should target cardiac rehabilitation participation to improve postoperative outcomes long beyond enrollment in a prehabilitation program. Additional beneficial postoperative behaviors, including sustained smoking cessation and the maintenance of a healthy BMI, should also be targets of future prehabilitation programs.

## 4. Prehabilitation in Cardiac Surgery 

While we have covered multiple studies investigating the role of prehabilitation through the perspective of perioperative frailty and the components of prehabilitation programs, this section is dedicated to analyzing the available published data and insights into ongoing trials specific to cardiac prehabilitation. 

In a single-arm pilot study, 22 patients older than 65 years old, with surgical waiting times of 6 weeks or longer, were recruited for prehabilitation before surgery; both open and endovascular cardiac procedures were selected for inclusion. The exercise regimen was unsupervised and consisted of incremental mobility and strength exercises, titrated to the exertion rating and exacerbation of the participant’s cardiac symptoms [28]. At baseline, the participants had a mean clinical frailty scale of 4.6 (vulnerable to mildly frail) and a mean Duke Activity Status Index (DASI) of 25 (equivalent to 5.8 metabolic equivalents). Twenty of the patients reported completing the exercise program at least thrice per week; fifteen attended the follow-up evaluation. At follow-up, the patients had significantly lower frailty scores as well as better performances on the Short Physical Performance Battery. After the prehabilitation, the patients had significantly better 6-min walk test (6MWT) performances, but the DASI scores were comparable. The mean length of stay was 5.8 days; all patients were discharged to home. 

In an ongoing clinical trial of perioperative optimization for elective cardiac surgery, participants are being randomized to either standard perioperative care or multimodal prehabilitation, followed by postoperative rehabilitation [59]. The prehabilitation program spans a minimum duration of 3 weeks, with three sessions each week. Each session includes 25 min of aerobic exercise, targeted at a low exertion intensity; 1–3 cycles of strength exercise, targeted at 50–80% of the 1 rep max; and supervised inspiratory muscle training exercise at 60–80% of the maximum inspiratory pressure (MIP), as well as guided breathing and relaxation exercises. In addition, the participants also receive group education sessions on diet and psychological coping strategies, with individual follow-up sessions offered as indicated. Lastly, those who currently smoke are offered weekly smoking cessation interviews. 

Those researchers conducted an exploratory analysis of the pilot study; 91 patients randomized to the multimodal prehabilitation were compared with 789 historical controls [60]. The cases were propensity-score-matched based on age; gender; body mass index; past medical history, including cardiac function; and procedural characteristics, including the length of surgery and total length of stay. In the matched cohort, patients who underwent prehabilitation had a significantly lower incidence of postoperative atrial fibrillation or flutter. There were no other differences in the incidence of other complications, prolonged mechanical ventilation, or 30-day mortality. 

In an earlier clinical trial, patients undergoing elective CABGs were randomized to 10 weeks of prehabilitation versus standard care [26]. The prehabilitation program consisted of twice-weekly sessions of aerobic exercises titrated to 40–70% of the participant’s capacity. In conjunction, they also received two supportive educational sessions during the same period, as well as a monthly call with the nurse clinician. Those authors reported that the patients in the intervention group had significantly shorter lengths of stay in the ICU (2 h less) and in the hospital (1 day less); they also reported better physical functioning in the intervention group but no difference in postoperative anxiety level. 

A clinical trial of all elective cardiac surgeries randomized 180 participants to receive routine care or 4 weeks of prehabilitation. The exclusion criteria included aortic stenosis with presyncope, uncompensated heart failure, complete heart blockage without a pacemaker, and malignant arrhythmia. The exercise prehabilitation consisted of 60-min in-person sessions twice a week, in addition to daily home exercises. The in-person exercise sessions consisted of aerobic exercises, resistance training, and stretches supervised by physical therapists. The exercise goal was to achieve 60–70% of the calculated target heart rate and moderate subjective exertion (BORG score: 12 or 13) [76]. Additionally, the participants were prescribed 45 min per day of unsupervised exercise according to their fitness level, with a lower target intensity. The participants were also given an inspiratory muscle training device (POWERbreathe medic plus) set to 50% of their MIPs. They were instructed to use the device twice per day, with 36 repetitions on each session. 

Those authors reported that most participants were not frail (Rockwood frailty score: <4) and 71% of the prehabilitation participants fulfilled the minimum compliance (attending 50% of the in-person sessions). There was no significant difference in 6MWT performance between the prehabilitation and control participants. The patients in the intervention arm had significantly higher MIPs, which were maintained for up to 12 weeks after surgery. There were no significant differences in risk of complications or length of ICU and hospital stay. There were also no differences in hand grip strength, QoL, or anxiety and depression [22]. 

Another study of patients undergoing elective CABGs randomized participants to a regimen of supervised exercise protocol vs. standard care [77]. Patients who had left main stenosis >50%, angina with low intensity exercise (50 Watts), left ventricular ejection fraction <35%, and other significant cardiac diseases were excluded. The prehabilitation consisted of supervised aerobic exercise sessions thrice per week on a bicycle machine. The participants underwent a baseline submaximal cardiopulmonary exercise test; the exercise intensity was then set at a 70% maximum oxygen uptake (VO_2_max) [2].

The primary outcome was the change in VO_2_max over time, which was not significantly different between the control and prehabilitation arms. Those authors also proposed the inclusion of endothelial function (reactive hyperemic index, measured using an EndoPAT® device, Atlanta, GA, USA); this was, however, not reported. Patients in the prehabilitation arm were noted to perform better on the Timed-Up-and-Go test (day before surgery: 6.1 s vs. 7.3 s; 3 weeks after surgery: 6.4 s vs. 7.5 s; *p* < 0.01). There were no differences in the 6-min walk test performance nor the global QoL [2].

## 5. Enhanced Recovery after Cardiac Surgery

Enhanced recovery after surgery (ERAS) is an evidence-based interdisciplinary approach to perioperative care, the goals of which are to facilitate swift postoperative recuperation while minimizing surgical stress. The first ERAS program was introduced by a Danish group in 1997, led by Dr. Henrik Kehlet. Since then, 23 ERAS surgical subspecialty guidelines have been created, including bariatric, gynecology, liver transplant, and, recently, cardiac. The characteristic features of cardiac surgery, including sternotomy, cardiopulmonary bypass, and postoperative ICU requirements, have enabled the creation of the Enhance Recovery After Cardiac Surgery (ERACS) program to help patients overcome these unique challenges. 

Similar to other ERAS protocols, the ERACS program emphasizes multimodal pain control, including the engagement of regional anesthesia techniques such as serratus anterior plane (SAP) blocks, preoperative gabapentin, and intraoperative dexmedetomidine to reduce postoperative opioid consumption and the associated adverse effects, which are contrary to the mission of the ERACS program [58,78,79,80]. The ERACS program also shares components with other ERAS programs, including goal-directed fluid therapy. Cardiac surgeons, anesthesiologists, and intensivists often have the luxury of invasive hemodynamic monitoring with real-time physiological variables, such as the cardiac index, the stroke volume index, and cardiac filling pressures. Integrating this information into fluid status management has resulted in a reduction in composite 30-day mortality and major postoperative complications, including infection and prolonged hospital LOS, compared to fluid management using the heart rate, central venous oxygen saturation, lactate levels, hematocrit values, and urinary output [81]. 

Multiple well-established factors, including surgical stress hyperglycemia and cardiopulmonary bypass, play roles in elevating blood glucose levels during and after cardiac surgery [82,83]. Inadequate postoperative blood glucose management has been associated with a myriad of complications across multiple organ systems, including cardiac, respiratory, immune, renal, neurological, and gastrointestinal sequala [84,85,86,87,88]. Patients with blood glucose levels above 250 mg/dL experienced an in-hospital mortality rate five times higher than those with blood glucose levels below 200 mg/dL [85]. A more recent study found that a blood glucose target of 160–200 mg/dL is associated with an almost seven times higher in-hospital mortality rate compared with a target of 120–160 mg/dL [89]. Collectively, the above-mentioned studies underscore the importance of perioperative glycemic control as a critical component of the ERACS program. Fortunately, technological advances will provide innovative ways to strengthen ERACS programs, such as automated insulin supply devices assisting and overseen by anesthesiologists [90].

Prehabilitation is also a part of the comprehensive preoperative care provided through the ERACS program. At the time of the ERACS guideline publication in 2019, a moderate-strength recommendation for prehabilitation in patients with multiple comorbidities and undergoing elective cardiac surgery was backed by moderate-quality evidence. However, in multiple retrospective studies that followed the implementation of the ERACS guidelines, prehabilitation was limited to education and encouragement without structured nutrition or supervised exercise programs, if prehabilitation was even included in the real-world ERACS programs [91,92,93]. The logistical difficulties in practically incorporating prehabilitation into the ERACS program were echoed in the 2024 Joint Consensus Statement by the ERAS Cardiac Society, the ERAS International Society, and The Society of Thoracic Surgeons when they also concluded that prehabilitation may be considered to optimize patients before nonurgent cardiac surgery, but with a low quality of evidence to support their recommendation [94]. The authors came to their joint-consensus conclusion on prehabilitation based on the abundant degree of variability between prehabilitation implementation, feasibility, and selected components. A standardized execution of prehabilitation with guided physical, nutritional, and psychological components may be crucial to detecting the true value prehabilitation brings to patients undergoing cardiac surgery. 

## 6. Conclusions

Prehabilitation prior to cardiac surgery may promote significant postoperative benefits in a vulnerable population preparing for major surgery. Unfortunately, the heterogenicity of the current literature supporting prehabilitation precludes a detailed evidenced-based prehabilitation structure. With each prehabilitation trial, a new permutation of prehabilitation appears; for example, a prehabilitation program consisting of CBT, dietitian-formulated meal plans, and thrice-weekly supervised aerobic exercise has yet to be implemented before cardiac surgery. This hypothetical program could display additive or even synergistic effects among the prehabilitation components that have not yet been recorded in the literature. 

Future clinical research should aim to incorporate components that have been previously investigated but in different combinations or with improvements to the interventions. For instance, a study can combine Sawatzky et al.’s 2014 supervised aerobic exercise protocol with Hartog et al.’s 2021 nutrition component and Dao et al.’s psychobehavioral component to include at least one intervention per prehabilitation component. With a large enough study, researchers can isolate the effects of each intervention to better guide clinicians in serving patients.

**Figure 1:**
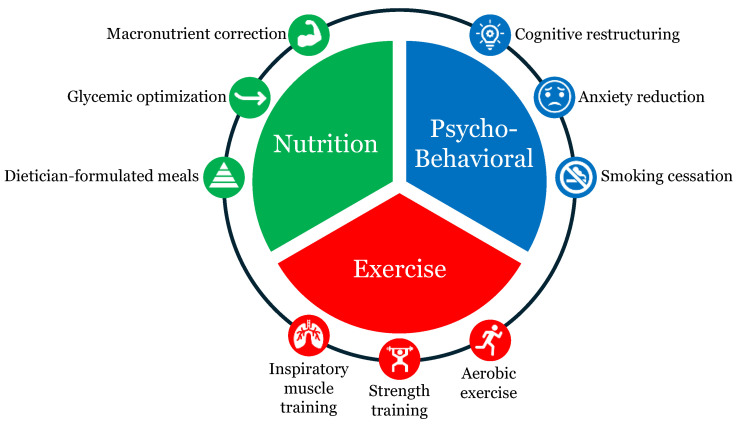
The three major cardiac prehabilitation components are nutrition, exercise, and psychobehavioral support, each with three specific interventions trialed in the literature.

Until more sophisticated literature is published, perioperative care team members, especially cardiac anesthesiologists, cardiac surgeons, and anesthesiologists specializing in perioperative medicine, should reflect on the existing body of literature to help craft an effective prehabilitation program. We believe that an ideal cardiac prehabilitation program is an individualized, patient-centered program that provides patients with as many interventions as possible from the three major components of prehabilitation—exercise, nutritional, and psychobehavioral (see Figure 1). With each component and intervention added into a prehabilitation regimen, the chances of improving the physiological reserves from one or more body systems increase. A cardiac prehabilitation program comprising only one or two components, with limited interventions from each component, may not produce statistically or clinically significant changes compared to the current standard of care. Our ideal cardiac prehabilitation program facilitates strengthening a patient’s physiological reserve across multiple organ systems, laying a resilient foundation for a meaningful recovery. 

## Figures and Tables

**Table 1:** Published prehabilitation exercise interventions, protocols, and outcomes.

Exercise Intervention	Author (Year)	Intervention Protocol	Outcome
Aerobic exercise (Cycle ergometer, treadmill, walking)	Arthur et al. (2000) [26]	Supervised aerobics, 40–70% max heart rate, 90 min 2×/week	Decreased hospital LOS, improved QoL
	Rosenfeldt et al. (2011) [36]	Aerobics, 50% VO_2_max, one hour, supervised 2×/week for ×2 weeks, then 30 min 4×/week at home	No difference in LOS or QoL
	Tung et al. (2012) [37]	Supervised aerobics, 50–60% VO_2_max, one-hour 2×/week for 2 weeks	Decreased noninvasive ventilation and time to ambulation, improved QoL
	Sawatzky et al. (2014) [38]	Supervised aerobics, 85% max capacity, one-hour 2×/week until surgery, mean of 8 weeks	Improved 6-min walk test and gait speed, no difference in LOS
	Steinmetz et al. (2020) [2]	Supervised aerobics, 70% peak oxygen uptake, two 10-min workouts with 15 min of light gymnastics for 2 weeks	Improved 6-min walk test and Timed-Up-and-Go time, improved QoL
	Akowuah et al. (2023) [22] *	Aerobic and respiratory muscle training, two supervised one-hour sessions/week ×4 weeks, home exercise 45 minutes daily, 2×/day incentive spirometer	No difference in 6-min walk test or postoperative mortality, improved maximal inspiratory pressure
Strength training (Rowing, wall push-ups, heel raises, bicep curls, sit-to-stand)	Waite et al. (2017) [28]	Strength and balance, home, 3×/week	Reduced frailty, improved 6-min walk test and functional capacity
Respiratory muscle training (Incentive spirometry, deep breathing, forced expiration)	Hulzebos et al. (2006) [32]	Respiratory muscle, 20 min/day, supervised weekly, mean of 8 weeks	Reduced respiratory complications, reduced pneumonia, shorter LOS
	Savci et al. (2011) [34]	Respiratory muscle, 30 min 2×/day, 5 days prior and 5 days after surgery	Increased strength, improved 6-min walk test, decreased ICU LOS
	Sobrinho et al. (2014) [35]	Respiratory muscle, supervised once daily until surgery	Shorter LOS, improved respiratory mechanics

* Mixed intervention consisting of aerobic exercise and respiratory muscle training. Abbreviations: length of stay (LOS), quality of life (QoL), maximal oxygen consumption (VO_2_max), ICU (intensive care unit).

## Data Availability

No new data were created or analyzed in this study. Data sharing is not applicable to this article.
